# What are the most effective techniques in changing obese individuals’ physical activity self-efficacy and behaviour: a systematic review and meta-analysis

**DOI:** 10.1186/1479-5868-10-29

**Published:** 2013-03-03

**Authors:** Ellinor K Olander, Helen Fletcher, Stefanie Williams, Lou Atkinson, Andrew Turner, David P French

**Affiliations:** 1 Applied Research Centre in Health and Lifestyle Interventions, Coventry University, Priory Street, Coventry, CV1 5FB, UK; 2 School of Psychological Sciences, University of Manchester, Oxford Road, Manchester M13 9PL, UK

**Keywords:** Obesity, Self-efficacy, Physical activity, Behaviour change techniques

## Abstract

Increasing self-efficacy is generally considered to be an important mediator of the effects of physical activity interventions. A previous review identified which behaviour change techniques (BCTs) were associated with increases in self-efficacy and physical activity for healthy non-obese adults. The aim of the current review was to identify which BCTs increase the self-efficacy and physical activity behaviour of obese adults. A systematic search identified 61 comparisons with obese adults reporting changes in self-efficacy towards engaging in physical activity following interventions. Of those comparisons, 42 also reported changes in physical activity behaviour. All intervention descriptions were coded using Michie et al’s (2011) 40 item CALO-RE taxonomy of BCTs. Meta-analysis was conducted with moderator analyses to examine the association between whether or not each BCT was included in interventions, and size of changes in both self-efficacy and physical activity behaviour. Overall, a small effect of the interventions was found on self-efficacy (*d* = 0.23, 95% confidence interval (CI): 0.16-0.29, p < 0.001) and a medium sized effect on physical activity behaviour (*d* = 0.50, 95% CI 0.38-0.63, p < 0.001). Four BCTs were significantly associated with positive changes in self-efficacy; ‘action planning’, ‘time management’, ‘prompt self-monitoring of behavioural outcome’ and ‘plan social support/social change’. These latter two BCTs were also associated with positive changes in physical activity. An additional 19 BCTs were associated with positive changes in physical activity. The largest effects for physical activity were found where interventions contained ‘teach to use prompts/cues’, ‘prompt practice’ or ‘prompt rewards contingent on effort or progress towards behaviour’. Overall, a non-significant relationship was found between change in self-efficacy and change in physical activity (Spearman’s Rho = −0.18 p = 0.72). In summary, the majority of techniques increased physical activity behaviour, without having discernible effects on self-efficacy. Only two BCTs were associated with positive changes in both physical activity self-efficacy and behaviour. This is in contrast to the earlier review which found a strong relationship between changes in physical activity self-efficacy and behaviour. Mechanisms other than self-efficacy may be more important for increasing the physical activity of obese individuals compared with non-obese individuals.

## Introduction

Approximately 200 million men and 300 million women are currently obese worldwide [1], with prevalence increasing [2]. Obesity is associated with numerous health risks, including an elevated risk of diabetes [3], heart failure [4], and depression [5]. Consequently, it has been argued that obesity is now the second largest modifiable cause of preventable death [6]. To alleviate these health risks in obese adults, physical activity has been recommended [7].

Self-efficacy has been identified as a key determinant in increasing physical activity [8]. Self-efficacy is the belief that one has the ability to successfully engage in a specific behaviour, such as physical activity. Findings from experimental studies show that self-efficacy can mediate the effects of interventions on physical activity behaviour. For example, Darker and colleagues found that the participants who showed largest changes in walking self-efficacy following a single session walking intervention were also the ones who showed the largest increases in objectively assessed walking behaviour [9].

Given that self-efficacy for physical activity is an important determinant of physical activity, it becomes essential to identify the best methods of increasing self-efficacy for physical activity. A recent systematic review and meta-analysis identified which behaviour change techniques (BCTs) were associated with an increase in self-efficacy for physical activity and physical activity behaviour [10,11]. This review identified intervention studies targeting physical activity in ‘healthy’ adults that also measured self-efficacy for physical activity. All interventions were coded using a standardised taxonomy of behaviour change techniques [12] to assess which BCTs were present in each intervention. Small significant effects of interventions were found on self-efficacy (*d* = 0.16, 95% confidence interval [CI]: 0.08-0.24, P < .001) and physical activity (*d =* 0.21, 95% CI 0.11-0.31, P < 0.001) and a significant large relationship between change in self-efficacy and change in physical activity behaviour was observed (Spearman’s Rho = 0.69, p < 0.001) [10]. Three BCTs were associated with significant increases in both self-efficacy and physical activity behaviour; ‘action planning’, ‘reinforcing effort or progress towards behaviour’ and ‘provide instruction’. These findings are important as they provide researchers as well as practitioners with information regarding which intervention components may increase intervention efficacy [12].

However, the Williams and French [10] review only included ‘healthy’ (i.e. participant groups not characterised by a common diagnosis) and non-obese individuals (BMI <30) and it is uncertain if the same BCTs would be effective in increasing self-efficacy and physical activity behaviour in other populations. For example, another recent systematic review did not find any effective behaviour change techniques for changing physical activity behaviour in obese adults with obesity-related co-morbidities or risk factors [13], suggesting that different BCTs may be effective at changing physical activity in different populations.

The aim of the present review was to identify which behaviour change techniques were associated with increases or decreases in self-efficacy for physical activity and physical activity behaviour in obese adults. A secondary aim of this review was to assess if the same techniques which were effective at changing self-efficacy were also effective at changing physical activity in this population.

## Methods

### Inclusion criteria

Eligible study designs included randomised controlled trials, non-randomised controlled trials, quasi-experimental studies or studies with pre-post design. Studies which used qualitative methods, a correlational design or used self-efficacy as a predictor only were excluded. Only English language reports were included for pragmatic reasons.

To be included in the review, the sample had to have a mean BMI of 30 or above (i.e. obese) and a mean of 18 years or more.

One of the intervention aims had to be to increase physical activity. Hence interventions which aimed to alter physical activity and eating behaviour were included. Interventions that focused on sport performance or were laboratory-based and did not aim to increase frequency or duration of physical activity behaviour were excluded.

All included studies had to report an experimentally induced change in physical activity self-efficacy. That is, physical activity self-efficacy had to be measured pre and post intervention when there was no comparison group or be measured for both intervention and comparison groups at least once following the end of the intervention. Where identified studies otherwise satisfied the inclusion criteria, but the report lacked this self-efficacy data the corresponding author was contacted for additional information.

### Search method

**Figure 1:**
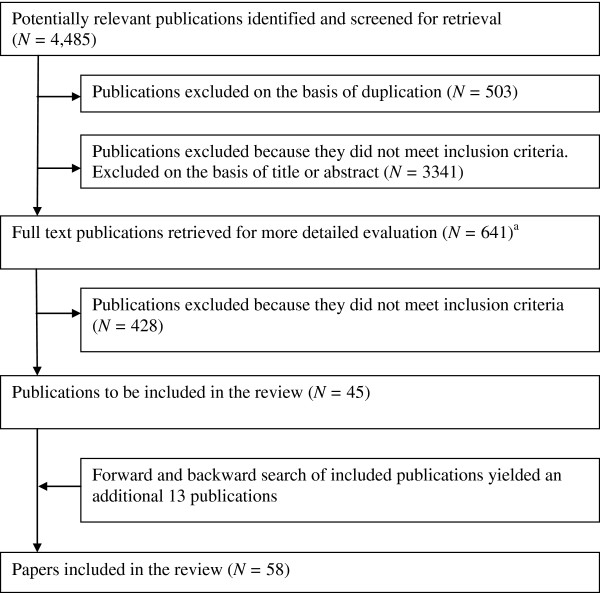
**Flowchart describing the number of articles retrieved, and included and excluded at each stage of the review process. **^a^This number includes studies that were retrieved with another search criteria in mind see [17].^1^

The electronic databases PsycInfo (1966–2011) and Scopus (1960–2011) were searched using a broad search strategy including self-efficacy, physical activity and trial terms (see Appendix 1). An initial search was conducted in June 2011 and updated in November 2011. All searches and eligibility assessment were conducted by the first and fourth author, through first screening of abstracts and subsequent examination of full texts where appropriate. All included papers were also subjected to forward and backward searches. See Figure 1 for a flowchart illustrating the review process.

### Data extraction

Relevant papers were entered into EndNote X3, and study and intervention characteristics as well as sample sizes, means and standard deviations were extracted by the first author. Effect size estimates (standardised mean difference or Cohen’s *d*[14]), were calculated by the same author. All intervention descriptions were double coded for behaviour change techniques using the Coventry Aberdeen LOndon REfined (CALO-RE) taxonomy [12] by the first author and either the second or third author. The CALO-RE taxonomy is an updated and expanded version of the taxonomy developed by Abraham and Michie [15], and was developed to identify BCTs used in physical activity or healthy eating interventions. Interrater reliability as assessed by kappa, corrected for chance agreement, was 0.68. This was calculated based on double coding of 23% of the intervention descriptions. Any disagreements between coders were resolved by discussion.

### Data analysis

The effect sizes and meta-analyses for self-efficacy and physical activity were conducted separately. The meta-analytic calculations were performed using Schwarzer’s (1987) Meta computer program [16], using a random-effects model. When studies reported more than one experimental group, each experimental group was compared to the control group to yield effect size estimates. When a study reported data at several time points post intervention, the one most immediately after the intervention end was used as this is when the largest effect attributable to the intervention should have occurred. When a comparison group had a mean BMI below 30, baseline and post intervention scores for the intervention group (with a BMI above 30) were analysed as a pre-post design. Heterogeneity was assessed using the *Q* coefficient. Moderator analyses investigated causes of heterogeneity, by comparing effect size estimates for groups of studies characterised by the presence or not of each behaviour change technique. Pairwise Z tests were conducted for each intervention technique to assess whether two groups had significantly different effect size estimates.

Spearman’s Rho correlation coefficient was used to assess whether change in self-efficacy was associated with change in physical activity behaviour. For each BCT the effect size estimate where the technique was not present was subtracted from the effect size estimate where the technique was present to calculate a difference score for both self-efficacy and physical activity behaviour. These differences were then correlated across BCTs.

## Results

The electronic search identified 4485 potential publications, of which 641 were retrieved for full text examination (some of these were retrieved with another search criteria in mind see [17])^1^. Of those, data from 45 publications were included in the review. Forward and backward searches identified another 13 eligible publications (see Figure 1). A total of 61 comparisons (58 publications) were included for the self-efficacy analyses [18-75] and 42 comparisons (39 publications either linked to or the same as the original 58 publications) were included for the physical activity behaviour analyses [18,19,24-26,28,35,36,38,40-43],[45,47-50,52,54,56-59,61,63,65-68],[70,71,73,75-81].

### Study characteristics

The mean number of participants in the comparisons included in the self-efficacy analysis was 181 and 170 (range 21 to 860 for both analyses) for the studies only included in the physical activity behaviour analysis. Overall, 25 of the comparisons employed a controlled design, and 36 used a pre-post design (see Table 1). Barrier self-efficacy was most commonly assessed (77% of all comparisons), with task self-efficacy assessed in 14% of comparisons (see Table 1).

**Table 1:** Summary of the participant and study characteristics of included publications

***Participant characteristics***^**a**^	***Mean (standard deviation) for self-efficacy analysis***	***Mean (standard deviation) for physical activity analysis***
Mean age in years of participants (range 28–77 years)^a^	49.1 (9.5)	50.0 (10.0)
Mean BMI of participants (range 30–42)^a, b^	34.5 (3.7)	34.5 (3.9)
Mean percentage of females per study (range 0-100%)^a, b^	79.2% (29.7)	73.1% (32.7)
Mean percentage of white participants per study (range 0-100%)^a, b^	59.6% (31.3)	59.0% (36.3)
*Study design*^*c*^	*Frequencies for self-efficacy analysis*	*Frequencies for physical activity analysis*
Controlled trials	25	18
Pre-post design	36	24
*Type of self-efficacy measured*^*d*^	*Frequencies for self-efficacy analysis*	*Frequencies for physical activity analysis*
Task self-efficacy	9	N/A
Barrier self-efficacy	47	N/A
Combined barrier and task self-efficacy	1	N/A
Other/unclear	3	N/A

^a^ Data on age, exact BMI, gender and ethnicity was not provided by all studies.

^b^ This is the range for both the self-efficacy and physical activity studies.

^c^ This data is for the statistical analyses conducted, some of the studies were RCT’s but were analysed as pre-post studies.

^d^ One study measured perceived behavioural control, not self-efficacy [68].

### Intervention characteristics

Despite assessing self-efficacy, an explicit theoretical basis was mentioned for only two thirds of studies, with the most frequent being Social Cognitive Theory [82] (see Table 2). Interventions were delivered by a wide variety of people and in a variety of locations, but most commonly a health and fitness professional in a fitness centre or gym (see Table 2). Almost half of interventions had an explicit focus on weight loss or weight maintenance, and two thirds focused on healthy eating in addition to physical activity (see Table 2).

**Table 2:** Summary of intervention characteristics of included publications for self-efficacy analysis

***Intervention characteristics***	***Frequencies for self-efficacy analysis (k = 61)***	***Frequencies for physical activity analysis (k = 42)***
*Theoretical basis*		
Theoretical basis explicitly mentioned	41	26
Some theory mentioned	6	5
No theoretical basis explicitly mentioned	14	11
Social Cognitive Theory	40	26
Transtheoretical Model	2	1
Self-determination Theory	2	2
Other/Unclear	17	13
*Type of activities*		
Individual	26	17
Group	31	22
Both individual and group	4	3
*Intervention focus*		
Exercise (e.g. aerobics class, gym, jogging)	3	0
Lifestyle physical activity (e.g. gardening, walking etc.)	31	25
Weight loss/management	27	17
Intervention also includes healthy eating focus	43	29
*Delivered by*		
‘Facilitator’/’Interventionist’	8	8
Health and fitness professional	22	9
Nurse or GP	6	4
Peers/lay expert	4	4
Researcher	8	5
Not stated	5	4
Other (including coach, dietician, instructor)	8	8
*Setting*		
By internet/post/telephone	3	2
Church	2	2
College/University	4	1
Community Centre	6	6
Fitness centre/gym	20	6
GP Surgery/Hospital	5	4
Participants home	4	3
Workplace	1	1
Unclear/Other	16	17
*Delivery mode*		
Counselling session	33	20
Discussion Group	18	14
Telephone	3	2
Web-based	7	6

A mean of 10.5 (SD = 6.4) BCTs were identified for the 61 comparisons included in the self-efficacy analysis. The control group interventions had a mean of 0.8 BCTs (SD = 1.5). A mean of 9.0 (SD = 5.3) BCTs were identified for the 42 comparisons included in the physical activity behaviour analysis. The control group interventions had a mean of 0.7 BCTs (SD = 1.5). The most commonly used BCTs in both analyses were ‘goal setting (behaviour)’, ‘prompt self-monitoring of behaviour’ and ‘prompt practice’ (see Table 3).

**Table 3:** Frequencies of behaviour change techniques that were used in the interventions

***Technique***	***Self-efficacy (k = 61 comparisons)***		***Physical activity (k = 42 comparisons)***	
	**N**	**%**	**N**	**%**
5. Goal setting (behaviour)	48	78.7%	34	81%
16. Prompt self-monitoring of behaviour	45	73.8%	29	69%
26. Prompt practice	42	68.9%	27	64.3%
8. Barrier Identification/Problem solving	39	63.9%	24	57.1%
35. Relapse prevention/coping planning	38	62.3%	25	59.5%
21. Provide instruction on how to perform the behaviour	37	60.7%	22	52.4%
29. Plan social support/social change	34	55.7%	21	50%
1. Provide information on consequences of behaviour in general	33	54.1%	20	47.6%
2. Provide information on consequences of behaviour for the individual	30	49.2%	16	38.1%
9. Set graded tasks	28	45.9%	17	40.5%
10. Prompt review of behavioural goals	26	42.6%	14	33.3%
38. Time management	26	42.6%	16	38.1%
6. Goal Setting (outcome)	23	37.7%	12	28.6%
12. Prompt rewards contingent on effort or progress towards behaviour	23	37.7%	11	26.2%
19. Provide feedback on performance	23	37.7%	11	26.2%
33. Prompt self-talk	22	36.1%	11	26.2%
36. Stress Management/emotional control training	22	36.1%	12	28.6%
13. Provide rewards contingent on successful behaviour	19	31.1%	9	21.4%
23. Teach to use prompts/cues	18	29.5%	7	16.7%
25. Agree behavioural contract	17	27.9%	5	11.9%
7. Action planning	12	19.7%	7	16.7%
22. Model/demonstrate the behaviour	10	16.4%	9	21.4%
28. Facilitate social comparison	7	11.5%	6	14.3%
20. Provide information on where and when to perform the behaviour	4	6.6%	4	9.5%
37. Motivational interviewing	4	6.6%	3	7.1%
15. Prompting generalisation of a target behaviour	3	4.9%	3	7.1%
17. Prompt self-monitoring of behavioural outcome	2	3.3%	2	4.8%
27. Use of follow up prompts	2	3.3%	1	2.4%
11. Prompt review of outcome goals	1	1.6%	1	2.4%
18. Prompting focus on past success	1	1.6%	0	0%
24. Environmental restructuring	1	1.6%	0	0%
39. General communication skills training	1	1.6%	1	2.4%
3. Provide information about others’ approval	0	0%	0	0%
4. Provide normative information about others’ behaviour	0	0%	0	0%
14. Shaping	0	0%	0	0%
30. Prompt identification as role model/position advocate	0	0%	0	0%
31. Prompt anticipated regret	0	0%	0	0%
32. Fear arousal	0	0%	0	0%
34. Prompt use of imagery	0	0%	0	0%
40. Stimulate anticipation of future rewards	0	0%	0	0%

### Changes in self-efficacy

**Figure 2:**
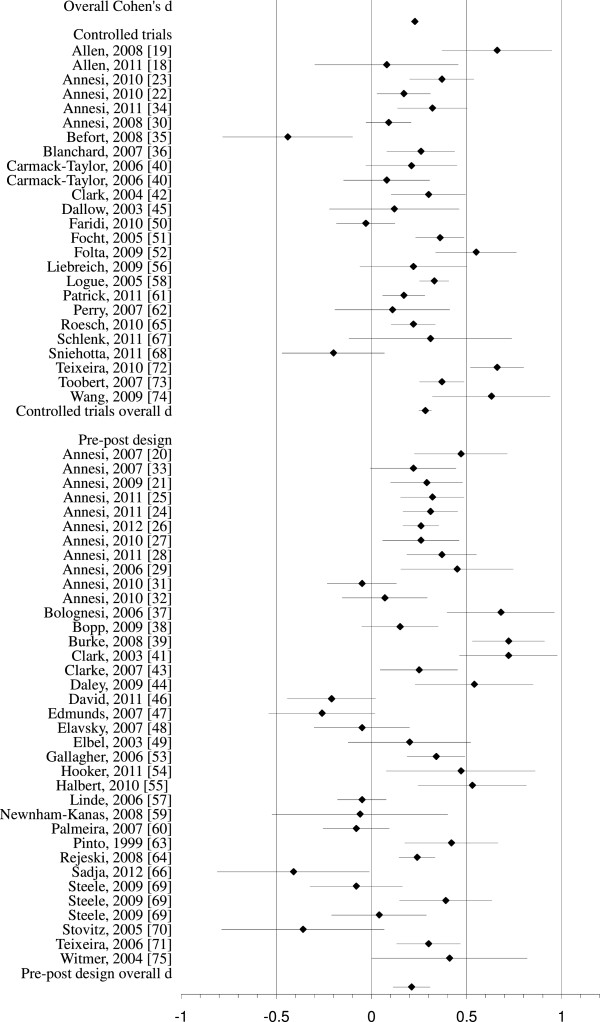
Forest plot showing self-efficacy effect sizes with 95% CI for each study, with studies ordered by reserach design.

For the analysis of changes in self-efficacy, 61 comparisons were included, indicating a small effect of the interventions on self-efficacy (*d* = 0.23, 95% confidence interval (CI): 0.16-0.29, p < 0.001). Fail-safe N (p = 0.05) was large: it would require an additional 2113 studies showing a zero effect not included in the present analysis for the relationship between interventions and self-efficacy to become statistically non significant [83]. A forest plot showing self-efficacy effect sizes with 95% CI for each study ordered by research design is given in Figure 2. A greater variability in effect size estimates existed than could be explained by random sampling error alone (*Q* = 129.27, p < 0.001). The amount of variance attributable to sampling error was 58.29%. Effect sizes for self-efficacy ranged from *d = −*0.44 [35] to *d =* 0.72 [39,41].

In total, 28 moderator analyses were conducted to investigate differences in self-efficacy according to presence or absence of BCTs (see Table 4). Moderator analyses were not conducted for those BCTs that were not coded as present in any (BCT: 3, 4, 14, 30, 31, 32, 34 and 40, as listed in Table 3) or in only one intervention group (BCT: 11, 18, 24 and 39, as listed in Table 3).

**Table 4:** Comparison between self-efficacy and physical activity behaviour, according to whether specific techniques are present in the physical activity intervention and when the technique is not present

***Technique***	***Self-efficacy***							***Physical activity***						
	***Present***			***Not present***				***Present***			***Not present***			
	***n***	***k***	***d***	***n***	***k***	***d***	***z***	***n***	***k***	***d***	***n***	***k***	***d***	***z***
1. Provide information on consequences of behaviour in general	5462	30	.174	4888	31	.206	.80	3721	19	.601	3893	23	.437	3.45***
2. Provide information on consequences of behaviour for the individual	4862	24	.244	5488	37	.213	.78	2544	10	.641	5070	32	.501	2.77**
5. Goal setting (behaviour)	7768	43	.212	2582	18	.268	1.22	5447	29	.624	2167	13	.346	5.31***
6. Goal Setting (outcome)	5514	21	.235	4836	40	.216	.48	3575	10	.751	4039	32	.448	6.31***
7. Action planning	1563	12	.322	8787	49	.208	2.05*	1026	7	.613	6588	35	.520	1.33
8. Barrier Identification/Problem solving	6496	38	.247	3404	23	.189	1.40	4617	23	.678	2997	19	.349	6.78***
9. Set graded tasks	5833	26	.167	4517	35	.287	3.03**	4315	17	.716	3299	25	.392	6.74***
10. Prompt review of behavioural goals	5610	26	.245	4740	35	.212	0.83	3596	14	.628	4018	28	.494	2.80**
12. Prompt rewards contingent on effort or progress towards behaviour	4312	23	.236	6038	38	.223	0.32	2407	11	.830	5207	31	.429	7.74***
13. Provide rewards contingent on successful behaviour	4420	19	.249	5930	42	.215	0.85	2624	9	.682	4990	33	.494	3.74***
15. Prompting generalisation of a target behaviour	598	3	0.05	9752	58	.237	2.20*	598	3	.380	7016	39	.552	1.96*
16. Prompt self-monitoring of behaviour	8552	43	.216	1798	18	.256	0.76	6294	29	.600	1320	13	.279	5.16***
17. Prompt self-monitoring of behavioural outcome	466	2	.468	9884	59	.217	2.59**	497	2	.804	7117	40	.524	2.85**
19. Provide feedback on performance	4795	23	.244	5555	38	.214	0.75	2804	11	.637	4810	31	.497	2.81**
20. Provide information on where and when to perform the behaviour	787	3	.309	9563	58	.224	1.13	815	3	.488	6799	39	.544	0.73
21. Provide instruction on how to perform the behaviour	5346	31	.241	5004	30	.213	0.70	3583	19	.676	4031	23	.430	5.15***
22. Model/demonstrate the behaviour	881	10	.155	9469	51	.235	1.12	841	9	.797	6773	33	.511	3.70***
23. Teach to use prompts/cues	3975	18	.236	6375	43	.221	0.37	2112	7	.949	5502	35	.433	9.50***
25. Agree behavioural contract	3782	17	.262	6568	44	.205	1.38	1823	5	.880	5791	37	.480	7.03***
26. Prompt practice	5713	35	.231	4637	26	.220	0.28	4071	25	.725	3543	17	.283	9.30***
27. Use of follow up prompts	334	2	.338	10016	59	.223	1.01	No interventions included this technique						
28. Facilitate social comparison	708	6	.176	9642	55	.232	0.71	446	5	.845	7168	37	.520	3.14***
29. Plan social support/social change	6144	32	.258	4206	29	.181	1.91*	3983	19	.689	3631	23	.388	6.36***
33. Prompt self-talk	4717	22	.232	5633	39	.222	0.25	2854	11	.751	4760	31	.449	6.10***
35. Relapse prevention/coping planning	7209	37	.244	3141	24	.175	1.60	5067	24	.656	2547	18	.366	5.77***
36. Stress Management/emotional control training	4782	23	.222	5568	38	.184	.96	2983	13	.678	4631	29	.414	5.41***
37. Motivational interviewing	389	4	.223	9961	57	.224	0.004	351	3	.384	7263	39	.513	1.15
38. Time management	4740	26	.272	5610	35	.192	2.01*	2386	16	.553	5228	26	.472	1.58

*p < 0.05, **p < 0.01, ***p < 0.001.

Four BCTs were significantly associated with higher self-efficacy effect sizes when present (all; p < .05); ‘action planning’, ‘prompt self-monitoring of behavioural outcome’, ‘plan social support/social change’ and ‘time management’. Two BCTs were significantly associated with lower self-efficacy effect sizes when present ‘set graded tasks’ and ‘prompting generalisation of a target behaviour’. The presence or absence of the remaining 23 behaviour change techniques was not significantly associated with self-efficacy effect size estimates (see Table 4).

### Changes in physical activity

**Figure 3:**
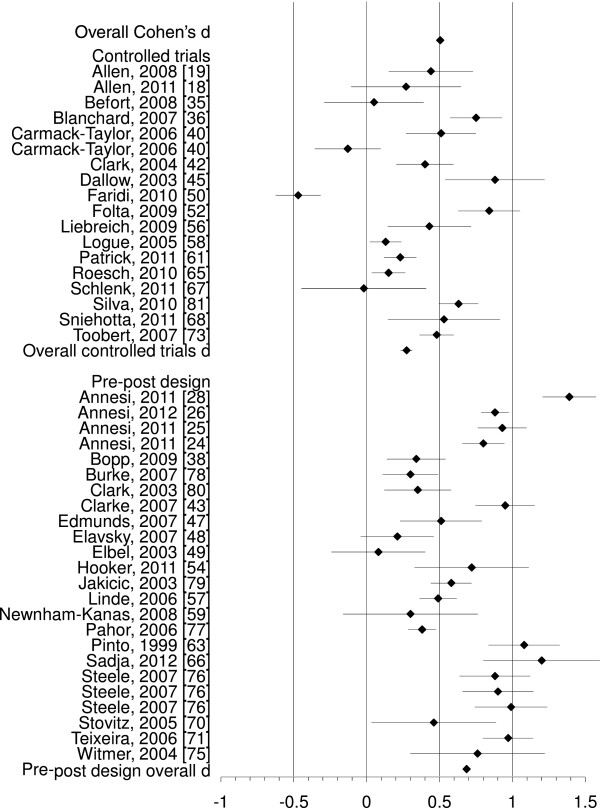
Forest plot showing physical activity effect sizes with 95% CI for each study, with studies ordered by research design.

For the analysis of changes in physical activity behaviour, 42 comparisons were included indicating a significant medium effect of the interventions on physical activity behaviour (*d* = 0.50, 95% CI 0.38-0.63, p < 0.001). Fail-safe N (p = 0.05) was large: it would require an additional 5789 studies showing a zero effect not included in the present analysis for the relationship between interventions and physical activity to become statistically non significant [83]. A forest plot showing physical activity effect sizes with 95% CI for each study ordered by research design is given in Figure 3. A greater variability in effect size estimates existed than could be explained by random sampling error alone (*Q* = 293.86, p < 0.001). The amount of variance explained by sampling error was notably lower than was the case for self-efficacy at 31.75%. Effect sizes ranged from *d = −*0.47 [50] to *d =* 1.2 [66].

In total, 27 moderator analyses were conducted to investigate differences in physical activity behaviour according to presence or absence of BCTs. Moderator analyses were not conducted for those BCTs which were not coded as present in any (BCT: 3, 4, 14, 27, 30, 31, 32, 34 and 40 as listed in Table 3) or in only one intervention group (BCT: 11, 18, 24 and 39 as listed in Table 3).

Twenty-one BCTs were significantly associated with higher physical activity behaviour effect sizes when present, and only ‘prompting generalisation of a target behaviour’ was associated with a lower effect size estimate when present (see Table 4). The greatest difference in effect size occurred when the following techniques were present; ‘teach to use prompts/cues’, ‘prompt practice’ and ‘prompt rewards contingent on effort or progress towards behaviour’. There were no significant differences in physical activity effect size estimates between interventions that included the remaining four BCTs and interventions that did not.

### Comparison of techniques associated with self-efficacy and physical activity

A negative non-significant relationship was found between the change in self-efficacy and the change in physical activity for the 27 behaviour change techniques included in at least two interventions (Spearman’s Rho = −0.18 p = 0.72). Of the 27 techniques included in both moderator analyses, only six did not show an increase in effect size when the technique was present for physical activity and of these two were associated with an increase in self-efficacy.

## Discussion

This meta-analysis of physical activity interventions for obese adults found a small (*d* = 0.23) but significant effect of interventions on self-efficacy and a significant effect of interventions on physical activity behaviour (*d* = 0.50) of medium size. The moderator analyses identified four behaviour change techniques that were associated with a higher self-efficacy effect size estimate. Only two of these techniques; ‘prompt self-monitoring of behavioural outcome’ and ‘plan social support/social change’, were also associated with higher effect size estimate for physical activity behaviour. In addition, two techniques were found to be associated with a lower self-efficacy effect size estimates; ‘set graded tasks’ and ‘prompting generalisation of a target behaviour’. The latter technique was also associated with a lower physical activity behaviour effect size estimate. For physical activity behaviour, 21 techniques in total were found to be associated with a higher effect size estimate. The largest effects were found for ‘teach to use prompts/cues’, ‘prompt practice’ and ‘prompt rewards contingent on effort or progress towards behaviour’. The association between the changes in self-efficacy and physical activity behaviour was small and not statistically significant (Spearman’s Rho = −0.18).

### Strengths and limitations

There are several strengths of this systematic review and meta-analysis. Firstly, we conducted a systematic review using broad search terms to increase the probability of identifying all eligible publications, and which yielded a good sized (k = 61) evidence base. Secondly, we used the same methods and analysis as a previous review [10], allowing for a comparison of effective BCTs between ‘healthy’ non-obese adults and obese adults. Thirdly, intervention contents were reliably coded using a standardised taxonomy for BCT’s [12].

There are a few limitations associated with this review. There were numerous BCTs examined as independent moderators leading to a large number of comparisons conducted. Thus, it is entirely possible that some of the significant effects were identified due to chance alone: there was an inflation of risk of type 1 error. The analyses were based on identifying associations between interventions which contained specific BCTs and two outcome variables. It is entirely possible that some of these associations identified are due to confounding variables, i.e. characteristics of population, intervention other than BCTs or type of self-efficacy measured^2^. The current analyses also only examine the associations with presence or absence of BCTs, and do not take into account quality of BCT delivery or combinations of techniques. Interventions are rarely developed to test single factors, thus combinations of techniques were common and individual techniques cannot be tested. Moreover, it is possible that some techniques are more common to cluster than others, thus our findings should not be taken to mean that these techniques has these effects when used on their own. Unfortunately, our study sample is too small for reliably testing the combinations of techniques. This is something that needs further investigation in future research.

Furthermore, coding interventions was at times difficult due to the lack of precision and detail provided, as mentioned previously by other research groups [13]. Based on this, we were only able to code intervention techniques that were explicitly stated and strongly suggest that authors describe their interventions using terms from the behaviour change taxonomy in the future. Encouragingly, some researchers do this [26], which makes these type of reviews more accurate. Additionally, this review is concerned with summarising existing evidence, thereby generating new hypotheses for future research to test using experimental designs without such potential confounders. Lastly, more studies could have been included if the focus of this review had solely been on what BCTs increase physical activity [84]. However, a strength of this review is that it investigates both physical activity behaviour and self-efficacy which allows examination of theoretical determinants of physical activity in this population for whom physical activity should be a priority.

### Which behaviour change techniques are associated with changes in self-efficacy for physical activity and physical activity behaviour in obese adults?

This review adds to the current literature by identifying which behaviour change techniques are associated with changes in self-efficacy and physical activity behaviour in an *obese* population. Previous reviews have identified BCTs effective in increasing this behaviour in other populations [10,84] including obese individuals with additional risk factors [13]. Similarly, the previous review concerning which BCTs were associated with self-efficacy was conducted in an explicitly non-obese population [10].

Four behaviour change techniques were found to be associated with increased self-efficacy. These involved planning, prompting and practical skills. ‘Action planning’, involves planning where and when to act and in which situation and it seems likely that greater goal specification, i.e. knowing what to do where and when, may encourage the belief that engaging in physical activity is feasible. Similarly, time management is a practical skill that may increase individuals’ belief that they can perform the behaviour by helping them feel they can better control potential obstacles. Neither of these BCTs however were associated with an increase in physical activity behaviour.

‘Planning social support/social change’ i.e. planning how to elicit social support for the target behaviour from other individuals may also help people feel more in control over the performance of physical activity by receiving greater practical support with obstacles such as family or work commitments. This is supported by an association between the presence of this BCT and behaviour. In addition, feeling supported may help this population cope with setbacks and relapses in physical activity.

‘Prompting self-monitoring of behavioural outcome’, is defined as keeping a record of a specific outcome expected to be influenced by the behaviour change. In the two instances where this technique was identified, the outcome was weight loss [71,72]. It may be that self-monitoring one’s weight and seeing a change in weight enhanced the individuals’ feelings of being in control of physical activity, assuming they attributed any weight changes to their physical activity behaviour.

Two behaviour change techniques were associated with decreased self-efficacy; ‘set graded tasks’ and ‘prompting generalisation of a target behaviour’. The first technique involves breaking down the behaviour into smaller, more achievable tasks, and is thought to enable the individual to build on small successes [12]. ‘Prompting generalisation of a target behaviour’ encourages the individual to try the behaviour in a different setting/situation, after first mastering it in one situation [12]. Both of these BCTs are based on the idea of breaking overall behaviour change into smaller achievable goals. However, to participants these BCTs may make the goals seem large, unmanageable and unattainable, and possibly seem to involve ‘moving the goalposts’. Both of these techniques are used in skilled approaches such as cognitive behaviour therapy [85]. However, they may be poorly implemented within the studies included in this review, as many interventions were delivered by people such as fitness professionals that have not necessarily been trained to deliver behaviour change interventions. ‘Prompting generalisation of a target behaviour’ was the only technique that was associated with lower physical activity behaviour.

Overall, the most commonly used techniques were not found to be the techniques that may be most effective in increasing self-efficacy or physical activity (see Table 3 and 4). One of the potentially most effective BCTs was ‘teach to use prompts/cues’ and was used in only 16% of all physical activity comparisons. The second potentially most effective technique ‘prompt practice’ was identified in almost two thirds of all the interventions. It appears that the use of BCTs such as ‘teach to use prompts/cues’ and ‘prompt practice’ which involve prompting self regulation may potentially be particularly effective in helping obese individuals engage in physical activity. This finding is in line with a previous review of general physical activity interventions [84].

Another technique, ‘prompt rewards contingent on effort or progress towards behaviour’ involves the individual using self-reward or praise for attempts at achieving the behaviour. It may be that this population particularly needs encouragement as they try to change their physical activity behaviour. This is in line with the BCT ‘plan social support/social change’ which was associated with increased self-efficacy and physical activity.

### Are the same techniques which are associated with increased self-efficacy also associated with increased physical activity? Are they the same as in the review of non-obese adults?

A negative and non-significant association (rho = −0.18) between changes in self-efficacy and changes in physical activity was observed across BCTs. Of the 28 techniques in the moderator analysis, only three BCTs were associated with the same result (increase or decrease in effect size for when the technique was present/not present) for both self-efficacy and physical activity behaviour. Two of these techniques, ‘prompt self-monitoring of behavioural outcome’ and ‘plan social support/social change’, were associated with a higher effect size estimate when the intervention included this technique. The third technique, ‘prompting generalisation of a target behaviour’, was associated with a lower effect size estimate when the interventions included this technique for both self-efficacy and physical activity behaviour. The majority of techniques included in moderator analyses (19/28) were associated with larger physical activity behaviour effect sizes but not self-efficacy effect sizes.

Taken together, these findings clearly suggest that there are many other routes apart from increasing self-efficacy that can help obese adults become more physically active. There were larger changes brought about in physical activity than for self-efficacy. Also, more BCT’s were associated with increases in physical activity than increases in self-efficacy. The conclusion that self-efficacy is not the only route to behaviour change is in line with a recent review update which concluded that there is currently limited support for self-efficacy to act as a mediator of physical activity changes [86], in contrast to a commonly held view [8].

On the contrary, there may be something about an obese population that results in self-efficacy not being an important route to changing physical activity. The results of the present review stand in striking contrast to those of a previous review of non-obese adults, which found a strong and significant (r = 0.69) relationship between change in self-efficacy and change in physical activity behaviour.

Social cognitive theory does not propose that increasing self-efficacy will inevitably result in behaviour change [82]. The theory states that the effects of self-efficacy on behaviour will be moderated by outcome expectancies, i.e. beliefs that a particular behaviour will lead to a particular outcome. Where an individual believes that the behaviour will not lead to a valued outcome, self-efficacy will not motivate behaviour change. For example, an individual may believe they can drink fewer alcoholic drinks, but if they do not think the amount they are drinking is harmful, such self-efficacy will not result in less consumption. In terms of the present review, obese individuals may not believe that increasing their physical activity will lead to weight loss, which presumably would be a highly valued goal. There is evidence that the relationship between increased physical activity and weight loss is far from straightforward [87], so this would be a reasonable outcome expectancy for many obese people. Thus, this population may be convinced by an intervention that they can increase their physical activity, but if they were not convinced that this would result in the salient outcome of weight loss, it would not necessarily result in increased physical activity.

The techniques associated with increasing obese adults’ self-efficacy and physical activity were generally not the same as the BCTs associated with such change in non-obese adults. For self-efficacy, the current review identified four techniques that were associated with increasing adults’ self-efficacy where a review focusing on non-obese adults found three such techniques [10]. The only BCT that was found to be associated with increased self-efficacy in both populations was ‘action planning’ [10]. The current review identified 21 BCTs that were associated with increased physical activity behaviour, whilst the review that focused on non-obese adults identified six BCTs [10]. Out of these six BCTs, four techniques were found to be associated with an increase in physical activity in both non-obese and obese adults (‘provide information on consequences of behaviour in general’, ‘prompt rewards contingent on effort or progress towards behaviour’, ‘provide instruction on how to perform the behaviour’ and ‘facilitate social comparison’). These results highlight the importance of selecting appropriate BCTs for each population, and not assuming that BCTs will be uniformly effective, assuming these associations represent unique causal effects of each BCT.

### Implications and future directions

If the associations identified in this review are shown to reflect causal effects of BCTs on physical activity, future interventions with this population should be able to bring about change in physical activity using approximately half the techniques examined: most techniques appear to be effective. However, greater change is likely with techniques concerned with self-regulation, replicating previous findings with a general population [84]. Furthermore, this review has identified some possibly effective yet seldom used BCTs such as ‘teach to use prompts/cues’. We suggest future interventions include the BCTs that this review has identified as possibly effective, to maximize the intervention’s potential to be effective. Unlike interventions with non-obese adults [10], it does not seem to be important to specifically target obese individuals’ self-efficacy for physical activity in order to change their physical activity behaviour.

The present review has suggested a number of techniques are effective at increasing physical activity in obese individuals. Future research should test whether these associations reflect causal processes by using the present evidence base to develop interventions and then test their efficacy. Future research should also test whether increasing physical activity through increasing individuals’ self-efficacy is the best route to increase physical activity behaviour in this population. The current findings suggest that there are alternative mechanisms for increasing obese individuals’ physical activity behaviour, and there is a need for future research to identify these.

A strong test of the causal nature of the relationships identified in the present review, and a previous one involving non-obese adults [10] is also required. This would involve developing two interventions, each based on the BCTs identified as most associated with change in each population. A comparison would then be made of the relative efficacy of interventions which are ‘matched’ to the population for whom the intervention was developed, and ‘mismatched’ i.e. delivered to the other population.

## Conclusion

In summary, this review and meta-analysis has identified several behaviour change techniques that are associated with increased self-efficacy and physical activity. Some of these techniques supported previous findings from a review with healthy and overweight adults [10], whilst other techniques may be effective in an obese population only. Thus, to develop effective physical activity interventions it may be important to consider tailoring intervention techniques to populations to a greater extent than is commonly the case.

## Endnotes

^1^ This search aimed to identify studies with obese people and older (>60 years) adults. This was in line with objectives of the research commissioned by Macmillan Cancer Support [17]. Hence the number of publications retrieved reported here includes some that were retrieved with the older adults search criteria in mind.

^2^ In this review the effect sizes for task and barrier self-efficacy respectively was not significantly different (task *d =* 0.26, barrier *d =* 0.22, p = 0.23).

## Appendix 1

Scopus (1960 – 2011)

Terms in title, abstract or keyword.

Self-efficacy or Bandura or social cognitive theory.

OR

Theory of planned behaviour or theory of planned behavior or theory of reasoned action or perceived behavioural control or perceived behavioral control.

AND

Clinica* tria* or Randomised controlled tria* or Randomized controlled tria* or

Blind or Controlled clinical trial or Mask or Random allocation or Double blind method or Intervention or Evaluation or Progra* or Follow-up study or Experiment.

AND

Physical activity or exercise or fitness or exertion.

PsycInfo (1966–2011).

Search terms.

Self-efficacy or Bandura or social cognitive theory.

OR

Theory of planned behaviour or theory of planned behavior or theory of reasoned action or perceived behavioural control or perceived behavioral control.

AND

Clinica* tria* or Randomised controlled trial or Randomized controlled trial or.

Blind or Controlled clinical trial or Mask or Random allocation or Double blind method or Intervention or Evaluation or Progra* or Follow-up study.

AND

Physical activity or exercise or sport or fitness.

## Competing interests

The authors declare that they have no competing interests.

## Authors’ contributions

DPF had the original idea for this review, which he developed with the input of EKO and AT. EKO and DPF developed the review protocol and EKO conducted the literature searches. EKO and LA were responsible for the title and abstract screening and full text assessment (with help from DPF). EKO, HF and SW coded the interventions, and did the data extraction together with LA. EKO conducted all statistical analysis with support from DPF and wrote the manuscript with help from DPF. All authors read and approved the final manuscript.
